# Predicting Lexical Norms: A Comparison between a Word Association Model and Text-Based Word Co-occurrence Models

**DOI:** 10.5334/joc.50

**Published:** 2018-11-27

**Authors:** Hendrik Vankrunkelsven, Steven Verheyen, Gert Storms, Simon De Deyne

**Affiliations:** 1 Laboratory of Experimental Psychology, KU Leuven, BE; 2 Computational Cognitive Science Lab, University of Melbourne, AU

**Keywords:** word associations, k-nearest neighbors, lexical norms, affective word characteristics, concreteness, age of acquisition

## Abstract

In two studies we compare a distributional semantic model derived from word co-occurrences and a word association based model in their ability to predict properties that affect lexical processing. We focus on age of acquisition, concreteness, and three affective variables, namely valence, arousal, and dominance, since all these variables have been shown to be fundamental in word meaning. In both studies we use a model based on data obtained in a continued free word association task to predict these variables. In Study 1 we directly compare this model to a word co-occurrence model based on syntactic dependency relations to see which model is better at predicting the variables under scrutiny in Dutch. In Study 2 we replicate our findings in English and compare our results to those reported in the literature. In both studies we find the word association-based model fit to predict diverse word properties. Especially in the case of predicting affective word properties, we show that the association model is superior to the distributional model.

## Introduction

In the past forty years, theories of concept representation have concentrated predominantly on (proto)typicality (e.g., Hampton, 1979; Rosch & Mervis, 1975), category hierarchies (Murphy & Lassaline, 1997; Rosch, Mervis, Gray, Johnson, & Boyes-Braem, 1976), categorization (Mervis & Rosch, 1981) and category-based induction of unfamiliar features like ‘uses a particular enzyme’ (Osherson, Smith, Wilkie, López, & Shafir, 1990) and familiar features like ‘can bite through wire’ (Smith, Shafir, & Osherson, 1993). These theories remain mostly agnostic about the connotative aspects of meaning, namely that most words in the lexicon are to some extent determined by how positive or arousing they are. Around the time of the cognitive revolution, Osgood, Suci, and Tannenbaum (1957) showed in a series of analyses that three connotative factors contribute consistently to judgments related to the meaning of words. Their work showed that evaluation, potency, and activity (equivalent to, respectively, valence, dominance, and arousal) explain large proportions of the total variance in word meaning. Yet, the typical textbook treatment of semantic concepts nowadays largely ignores the affective aspects of word meaning. Murphy’s (2002) ‘Big book of concepts’, for instance, hardly deals with affective variables at all, explicitly stating that it is better to know ‘that cats meow and have whiskers than […] their potency and evaluation.’ (Murphy, 2002, p. 515).

This negligence of affective dimensions is in sharp contrast to a different literature that showed that emotionally charged concepts are processed differently from emotionally neutral concepts. Especially in the case of abstract words, valence seems to play a crucial role in the processing and representation of concepts (Kousta, Vigliocco, Vinson, Andrews, & Del Campo, 2011). Furthermore, in a study on the graded structure of adjective categories, it was found that about 83% of the variance in valence ratings of 360 adjectives was explained by a word association-based similarity space (De Deyne, Voorspoels, Verheyen, Navarro, & Storms, 2014). The evidence in favor of an important role for affective dimensions in semantics for a large variety of words places question marks over any model of word meaning in which such dimensions do not play a significant role.

An alternative approach to study word meaning draws upon the old idea that the meaning of a word is determined by the context in which it is used (e.g., Firth, 1968, Wittgenstein, 1953). In these lexico-semantic models, words with similar meanings occur in similar sentences, paragraphs, or documents. In contrast to classical theories of semantics that primarily focus on categories of concrete nouns, like animals or tools, lexico-semantic models generally capture word meaning at large. These models have been proven to be instrumental in several lines of research, ranging from purely theoretical questions, such as atypical word processing (e.g., Plaut, McClelland, Seidenberg, & Patterson, 1996) and the structure and acquisition of the mental lexicon in children (e.g., Landauer & Dumais, 1997), to more pragmatic issues, including second language learning (e.g., de Groot, 1995), text processing and expert system development (e.g., Aitchison, 2003).

An interesting recent development that extends the utility of these models is the prediction from word co-occurrences of connotative properties like valence or arousal and other semantic properties like concreteness or the age-of-acquisition of concepts (e.g., Bestgen & Vincze, 2012; Recchia & Louwerse, 2015). The implications of these studies go beyond the methodology of predicting new norms for a large variety of words, they also indicate that certain general semantic properties such as valence might be encoded through language.

Instead of using external measures such as word co-occurrences derived from natural language to learn something about the mental representation of meaning, subjective internal measures such as feature norms or word associations provide the most direct way to assess the content of these representations (e.g., Deese, 1965). While word associations reflect co-occurrence in language, this relation is not particularly strong. For example, in a recent study by Nematzadeh, Meyland, and Griffiths (2017) a variety of text-based models including recent topic and word embedding models were used to predict word associations, and across a variety of models the highest reported correlation was .27. This is not surprising since the primary role of language is communication and therefore text corpora might only provide us with indirect clues of how the mental lexicon is structured. Given the low correspondence between language and mental representations, it is not clear to what degree other semantic properties, like valence or arousal, are encoded in the mental lexicon and can be derived from subjective measures such as word associations. A priori, we might assume that subjective measures provide a better approximation of mental representations because of the shared processes involved in the word association generation and rating studies (see Jones, Todd & Hills, 2015). Alternatively, subjective measures are far more restricted in terms of the amount of data they encode because they typically only include a subset of the possible associates between words (e.g. Hofmann, Kuchinke, Biemann, Tamm, & Jacobs, 2011). As a consequence, it is not clear to what degree indirect subjective measures such as those derived from word associations provide useful estimates of general word covariates.

The goal of the present study is twofold. First, we will provide a more direct investigation of how measures based on word associations compare to measures derived from word co-occurrences in predicting connotative factors. Second, we go beyond connotative meaning by also investigating concreteness and age-of-acquisition, because these two variables have been previously implicated as structuring principles of the mental lexicon. Before explaining our approach, we will first briefly describe the findings and methods used in previous work on the global semantic structure of the mental lexicon.

### Global semantic structure in the mental lexicon

In an extensive research program, Osgood et al. (1957) introduced the semantic differential technique to investigate what factors determine connotative word meaning. In an impressive series of studies in a multitude of languages, they showed that large proportions of the total variance in the meaning of words is captured by valence (16% to 34% VAF; variance accounted for), dominance (7% to 8% VAF), and arousal (5% to 6% VAF). The external validity of these factors is supported by replications in many different domains, including brain imaging (Lane, Chua, & Dolan, 1999; Lang et al. 1998; Maddock, Garrett, & Buonocore, 2003; Mourão-Miranda et al. 2003), semantic categorization (Moffat, Siakaluk, Sidhu, & Pexman, 2015; Niedenthal, Halberstadt, & Innes-Ker, 1999), affective priming (Fazio, 2001; Klauer, 1997), word associations (Cramer, 1968; Isen, Johnson, Mertz, & Robinson, 1985), and word recognition reaction times (De Houwer, Crombez, Baeyens, & Hermans, 2001; Kuperman, Estes, Brysbaert, & Warriner, 2014).

Two other organizing factors of the mental lexicon have been considered in addition to its connotative structure. A first variable that is of crucial importance in word meaning is the level of abstractness/concreteness of the concept denoted by a word (Binder, Westbury, McKiernan, Possing, & Medler, 2005). It has long been known that concrete words are processed more easily than abstract words, a phenomenon called the concreteness effect. This advantage for concrete words shows up in a number of tasks, such as lexical decision, recall, recognition, etc. (Paivio, Walsh, & Bons, 1994; Schwanenflugel, Harnishfeger, & Stowe, 1988).

A second variable that has been argued to be important in the organization of the mental lexicon is the age at which the meaning of a word is acquired (Zevin & Seidenberg, 2002). An explanation for this age-of-acquisition (AoA) effect is that early acquired words are thought to provide the backbone of the mental lexicon, whereas later acquired information is considered less well embedded (e.g., Steyvers & Tenenbaum, 2005). Empirically, this has been demonstrated in a variety of lexical processing tasks that involve semantic access (e.g., Brysbaert & Ghyselinck, 2006). In a recent mega study, estimated AoA explained about 5% of the variance in lexical decision times when controlling for other variables such as word frequency (Kuperman, Stadthagen-Gonzalez, & Brysbaert, 2012).

Taken together, both the affective variables valence, arousal, and dominance, and the variables concreteness and AoA appear to affect the organization of the mental lexicon, which provides the motivation for including them in the current study.

### Prediction of word properties

Word co-occurrence models have recently been used in predicting diverse semantic word properties, including, valence, arousal, dominance, concreteness, and age of acquisition (e.g., Bestgen & Vincze, 2012; Mandera, Keuleers, & Brysbaert, 2015; Recchia & Louwerse, 2015). Their considerable success in predicting these norms suggests language as reflected in (written and spoken) text corpora encodes a variety of semantic word properties.

Older work on word associations has shown that affective variables are strongly encoded in the responses people give (Deese, 1965). Furthermore, more recent studies on network assortativity – the tendency of response congruency between cues and targets – has shown that affective factors of the cue word, but also concreteness, are a strong determinant of the corresponding properties of the associative response (Van Rensbergen, Storms, & De Deyne, 2015).

Because the present paper aims to directly compare a proposal based on word associations with the language-based models, we provide a detailed overview of the methods and findings of the above cited papers. In all these models, the general approach has been to infer properties of words based on how similar these words are to a training set of words for which these properties were known through rating studies. Typically, predictions for a target word are based on the average of its *k*-nearest neighbors (determined by similarity) for a property of interest.

A first example of this approach is a study by Bestgen and Vincze (2012). This work relied on latent semantic analysis (LSA, Landauer & Dumais, 1997) to derive similarities between words. LSA derives word co-occurrences from paragraphs in a large text corpus. Dimension reduction through singular value decomposition was used to reduce the sparsity of the word co-occurrence vectors by representing words in a low-dimensional vector space typically ranging between 300 and 1,000 dimensions. Similarity between the word pairs was then established by computing the cosine between the low-dimensional word vectors. The corpus in this study consisted of the General Reading up to 1st year college TASA corpus (Landauer, Foltz, & Laham, 1998). The training set consisted of 953 words from the corpus for which norms were available in ANEW (Affective Norms for English Words; Bradley & Lang, 1999). The quality of the predictions was assessed by making use of leave-one-out cross-validation. Varying the number of near neighbors, *k*, between 1 and 50, the highest correlations between human ratings and estimates were .71, .56, and .60 for valence, arousal, and dominance respectively.

In a somewhat similar fashion, Recchia and Louwerse (2015) used the Google Web 1 T 5-gram corpus (Brants & Franz, 2006) and computed the positive point-wise mutual information (PPMI) cosines between word vectors as a proximity measure. Their approach was slightly different in that co-occurrences were defined at the sentence-level instead of the document level. Recchia and Louwerse trained the data on the words contained in the Warriner norms (Warriner, Kuperman, & Brysbaert, 2013) but not in the ANEW (Bradley & Lang, 1999). As a test set they used words from the ANEW that can also be found in the Warriner norms. The results were slightly better than those reported by Bestgen and Vincze (2012): The highest resulting correlations between the estimates and ANEW for valence, arousal, and dominance were .74, .57, and .62 for values of *k* equal to 15, 40, and 60, respectively.

Finally, in contrast to the previous work, a recent study used word associations to predict rated affective and other lexico-semantic variables (Vankrunkelsven, Verheyen, De Deyne, & Storms, 2015). In this study, we extrapolated affective and lexico-semantic word properties from Moors et al. (2013) and Brysbaert, Stevens, De Deyne, Voorspoels, and Storms (2014) by training a model using a sample of 200 words to accurately predict these properties for all 3,500 remaining words in the data. It was shown that using word association data, higher correlations with human norm data could be obtained than reported in the previously mentioned studies that relied on text corpora, with values of .89, .76, .77, .67, and .81, for valence, arousal, dominance, AoA, and concreteness, respectively. In this study the cosine similarities between words (after applying a PPMI weighting scheme) were used to construct semantic spaces using multidimensional scaling (MDS; Borg & Groenen, 2005). Next, word properties were predicted using property fitting (Kruskal & Wish, 1978), that is, by finding the optimal property direction in these semantic spaces using the words in the training set, and projecting the words to be predicted on this optimal direction. Even using semantic spaces with a dimensionality as low as 2, some variables could already be well predicted (*r* = .58, .32, .21, .23, .70, for valence, arousal, dominance, AoA, and concreteness, respectively).

A systematic comparison of methods to extrapolate word properties using different language models was conducted by Mandera et al. (2015). They compared vector representations based on bag of words models, LSA and topic models, and representations derived from word co-occurrence counts and prediction models that learn word embeddings using a simple neural network (word2vec; Mikolov, Chen, Corrado, & Dean, 2013). Two extrapolation techniques were compared. In the first one, the data were split up in a training and test set. Word properties from the test set were then predicted by assigning the mean of the *k*-nearest neighbors (*k*-NN from here on) in the training set. The second technique Mandera et al. employed was based on the random forest procedure. This method creates several decision trees that maximize information about the variable that is predicted, using different random samples of the full dataset. In a next step, all these trees were merged to reduce the risk of overfitting. The best predictions of AoA, concreteness, arousal, dominance, and valence, were obtained by using word vectors from the skip-gram model and using *k*-NN to extrapolate. Correlations with the human ratings were .72, .80, .48, .60, and .69, for the previously mentioned variables, respectively.

Together, these findings suggest that specific combinations of word co-occurrence count models or prediction models with particular extrapolation methods can lead to reasonable good performance for the semantic variables that are the focus of attention in this paper. However, Mandera et al. (2015) did not include word association data in their comparison. It therefore remains to be seen how word association data fare in predicting these word properties, especially since the one study that relied on word association data to predict them did not make use of the *k*-NN method, which Mandera et al. showed to be the most effective extrapolation technique.

### Present studies

The main aim of this paper was to evaluate how a model based on word associations can account for diverse word properties and how such predictions compare with language-based models. In a first study, we directly compared these two types of models in predicting the variables valance, arousal, dominance, age of acquisition, and concreteness for a large number of Dutch words. We employed the *k*-NN procedure for both models. In the second study, we predicted the same variables for English words, making use of a word association-based model, and compared the resulting predictions to results of text-based models previously described in the literature.

## Study 1

In this study, we directly compared estimates derived from word association data and word co-occurrence data, using the same criterion variable, that is, the same list of words, as well as the same ratings. Except for the source of data, the methods used for predicting valence, arousal, dominance, AoA, and concreteness, were kept identical. Furthermore, to investigate the differences between the two model predictions, we also correlated the residuals of each model with the predictions of the other model.

### Method

**Materials.** We used the Dutch word co-occurrence model described in De Deyne, Verheyen, and Storms (2015). This model is similar to the one used by Recchia and Louwerse (2015) with one main difference: rather than tracking word co-occurrences in 5-grams, words that co-occur in specific syntactic dependency relations were used. The corpus consisted of three sources of data: text derived from newspapers and magazines (74%), less formal online text retrieved from internet web pages (25%), and spoken text retrieved from Dutch movies subtitles (1%). In totality, the corpus consisted of 79 million tokens. Syntactic word dependencies were used (e.g., subject – object pairs), as previous research indicated superior performance of such models to simple word co-occurrence models in synonymy extraction (Heylen, Peirsman, & Geeraerts, 2008; Padó & Lapata, 2007). Using lemma forms, each sentence was parsed to uncover the dependency structure of the different sentences and only the lemmas that appeared at least 60 times were used. The final corpus consisted of 157 million co-occurrence tokens and 103,842 different lemmas. Further details of the model can be found in De Deyne et al. (2015).

The Dutch word association data used to derive similarities between word pairs are described in De Deyne, Navarro, and Storms (2013). They consist of associations to more than 12.000 words collected from more than 70.000 participants from Flanders and the Netherlands. In this study, a continued free word association task was used in which participants gave the first three associations to a cue word that came to their mind. Thus, unlike in the well-known, but older, USF norms (Nelson, McEvoy, & Schreiber, 2004), participants were not instructed to only generate ‘meaningful’ associations. Personal context-specific responses and clang responses were allowed as well. For each cue, associations were gathered from at least 100 different participants, resulting in a minimum of 300 responses.

In line with previous work, only responses that also served as cue words were included, so that the cue by response matrix could be transformed into a cue by cue square matrix, with 12.566 cue words. Similarities were derived using the cosine measure after applying a PPMI weighting scheme to avoid overweighting high-frequency edges between words (De Deyne et al. 2015).

Norms for the semantic variables were taken from two main sources. Ratings of valence, arousal, dominance, and AoA were those gathered by Moors et al. (2013). Concreteness ratings were taken from Brysbaert, Stevens, et al. (2014). Except for AoA, these ratings were collected using 7-point Likert scales. Table 1 shows that all ratings were highly reliable.

**Table 1:** Information about the lexico-semantic norms used in Study 1 and 2: Amount of words, number of raters per word, and split-half reliabilities.

	Study 1			Study 2		

	Words	Raters	Reliability	Words	Raters	Reliability

Valence^a^	4,299	64	.99^d^	13,915	20	.91
Arousal^a^	4,299	64	.97^d^	13,915	20	.69
Dominance^a^	4,299	64	.96^d^	13,915	20	.77
AoA^b^	4,299	32	.97^d^	30,121	18+	.92
Concreteness^c^	30,070	15	.91–.93^d,e^	37,058	25+	–

^a^ Norms from Moors et al. (2013) for Study 1 and from Warriner et al. (2013) for Study 2. ^b^ Norms from Moors et al. (2013) for Study 1 and from Kuperman et al. (2012) for Study 2. ^c^ Norms from Brysbaert, Stevens, et al. (2014) for Study 1 and from Brysbaert, Warriner, and Kuperman (2014) for Study 2. ^d^ Spearman-Brown corrected split-half correlations calculated on 10,000 different randomizations of the participants. ^e^ Reliabilities of each of five lists of ca. 6,000 words were within this range.

Predictions of the Dutch norm scores of valence, arousal, dominance, AoA, and concreteness for words that were present in all data sets, 2,831 words in total, were obtained using *k*-NN. The predictions were cross-validated using a leave-one-out approach as in Bestgen and Vincze (2012).

**Procedure.** We predicted the semantic variable scores of each word that was available in all datasets from its *k-*NN, both for the association data and for the text data. The parameter *k* took all numerical values between 1 and 50; together with values of 60, 70, 80, 90, and 100. To assess the quality of the predicted variables, we correlated the obtained predictions with the human norm data.

### Results and discussion

**Figure 1:**
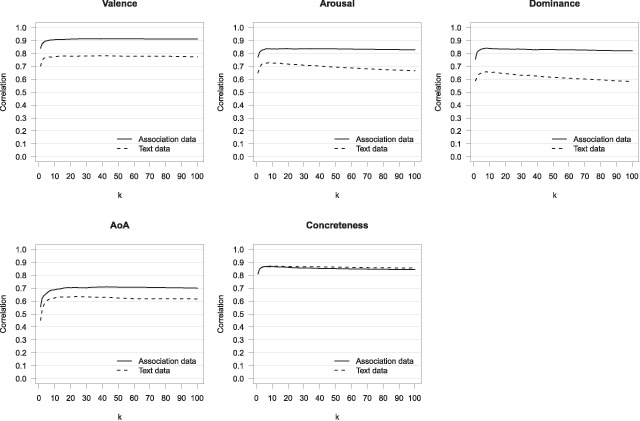
Correlations between predicted ratings and human ratings for valence, arousal, dominance, AoA, and concreteness, using association data or word co-occurrence data. Values of *k* are 1 to 50, 60, 70, 80, 90, and 100.

Figure 1 displays the correlations, as a function of the value of parameter *k*, between human and predicted ratings derived from either word association similarities or word co-occurrence similarities. As evident in the figure, the prediction of the affective variables using association data is superior to that derived from word co-occurrences. This was the case for all values of parameter *k*. Table 2 shows the highest correlation for each data source and variable. To test whether the corresponding correlations were significantly different we used the *cocor* package for R (Diedenhofen & Musch, 2015) and report the most conservative *p* values across various methods implemented in this package. The differences between the correlations of both data sources were all significant: .13 (*p* < .001), .11 (*p* < .001), and .18 (*p* < .001) for valence, arousal, and dominance, respectively. Predictions for AoA were also better using association data for every value of *k*, although to a lesser extent (see Figure 1).^1^ The difference between the highest correlations was .07 (*p* < .001). Concreteness was the only exception: the predictions from both data sources were on par for every value of *k* (see Figure 1). The difference between the highest correlations (see Table 2) was not significant (*p* = .59).

**Table 2:** The highest correlations and 95% confidence intervals for each variable per source of data (associations and text co-occurrences) using *k*-NN. All cross-validation correlations use the leave-one-out principle. The respective size of k is listed between square brackets.

		*k*-NN	

	*N*	Associations	Word co-occurrences

Valence	2,831	.91 (.91–.92) [50]	.78 (.77–.80) [38]
Arousal	2,831	.84 (.83–.85) [19]	.73 (.71–.75) [8]
Dominance	2,831	.84 (.83–.85) [8]	.66 (.64–.68) [8]
AoA	2,831	.71 (.69–.73) [43]	.64 (.61–.66) [24]
Concreteness	2,831	.87 (.86–.88) [10]	.87 (.86–.88) [11]

Although the association-based predictions outperformed the word co-occurrence-based predictions, some of the unexplained variance can be captured by the co-occurrence data and vice versa. We calculated the residuals of regression analyses with the human ratings as criteria and the predicted ratings as predictors. Using these residuals as criterion and the predictions of the other data source as predictors, we checked how much additional variance can be explained by the other data source. When using the association data residuals, the additional variance explained (*R^2^*) by the text data was .02, .03, .02, .08, and .06 (all *p*’s < .001), for valence, arousal, dominance, AoA, and concreteness, respectively. Using the word co-occurrence data residuals, the additional explained variance by the association data was .22, .18, .26, .17, and .06 (all *p*’s < .001).

Summarizing, a direct comparison of word associations and word co-occurrences as input data for predicting affective word variables, AoA, and concreteness, demonstrated clearly that the association data are well-suited to account for the predicted variables. Moreover, for all variables except concreteness, the prediction was better when using word association data.

## Study 2

In Study 2 we replicated the prediction of the lexico-semantic variables using English word association data. We used the same *k-*NN approach combined with leave-one-out validation as in Study 1. Study 2 is divided into two parts. In the first part, we used the largest available databases of norm scores for all the variables of interest (valence, arousal, dominance, AoA, concreteness). In the second part, we used the Affective Norms for English Words (ANEW; Bradley & Lang, 2017) to directly compare with the studies of Bestgen and Vincze (2012) and Recchia and Louwerse (2015). For these comparisons, predictions were derived for the same set of words used in these two papers.

### Method

**Materials.** To predict the variables of interest, similarities were derived in the same manner as in Study 1, but this time using word associations taken from the English Small World of Words project (SWOW-EN, English words; De Deyne, Navarro, Perfors, Brysbaert and Storms, 2018). The English word association data were gathered between 2011 and 2017 and consisted of associations to 12,292 words. In total, 88,710 English speaking participants from all over the world, but mainly from the US (53%), took part in a continued free word association task. Like in the Dutch project, they were asked to give the first three associations to a cue word that came to their mind. For every cue, associations were gathered from at least 100 different participants, resulting in a minimum of 300 associations (see De Deyne et al. 2018 for full details).

In the first part of the study, word ratings for valence, arousal, and dominance, were taken from Warriner et al. (2013), ratings for AoA from Kuperman et al. (2012) and ratings for concreteness from Brysbaert, Warriner, et al. (2014). Table 1 shows the important characteristics of these ratings, including the number of words, raters, and the obtained reliability. For the second part of this study, we used the ANEW which consist of 3,188 words at present, including the 1,034 words^2^ used by Bestgen and Vincze (2012) and the 2,327 words used by Recchia and Louwerse (2015) in earlier versions of the ANEW.

**Procedure.** As for Study 1, we predicted lexico-semantic variables using the *k-*NN method (with *k* ranging from 1 to 50, plus *k* values 60, 70, 80, 90, and 100) with leave-one-out cross-validation. In a first analysis (Part 1), we predicted scores for all words that were available in both the association dataset and in the lexical norms. These were 8,770 words for valence, arousal, and dominance, 10,032 for AoA, and 10,957 for concreteness. In a second analysis (Part 2a), we predicted the words from the ANEW dataset (Bradley & Lang, 1999) using the same method as Bestgen and Vincze (2012) which is the same as the one described above. There were 946 shared words in the association data and the ANEW, a value comparable to the 951 shared words in Bestgen and Vincze. We also performed a similar analysis (Part 2b) as Recchia and Louwerse (2015) did, based on the words in the first update of the ANEW (2,471 words) that are also present in the Warriner et al. (2013) norms as possible test data (i.e., 2,333 words). All words from Warriner et al. that are not scored in the ANEW (11,582 words) were used as possible training data. The overlap with the association data was 2,156 words for the test set, and 6,614 for the training set. For each word in the test set, we looked for the *k*-NN, in terms of association similarity, in the training set and estimated the word properties using the mean of the neighbors. The values of *k* were the same as mentioned above.

### Results and discussion

**Figure 2:**
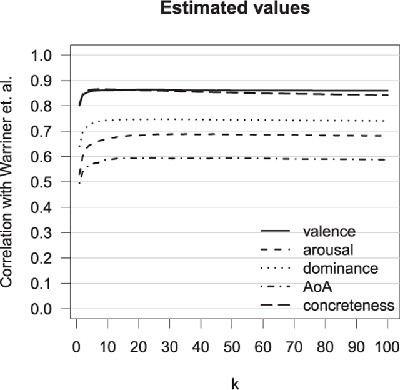
Correlations between estimated values based on the word association data and human ratings for valence, arousal, dominance, AoA, and concreteness. Values of *k* are 1 to 50, 60, 70, 80, 90, and 100.

**Part 1.** The results from the first analysis are shown in Figure 2 and Table 3. As in Study 1, we were again able to predict human ratings of affective variables quite well. The highest correlations obtained, with an optimal parameter *k*, were .86, .69, and .75 for valence, arousal, and dominance, respectively. These correlations were slightly lower than the correlations based on the association data observed in Study 1. A straightforward reason for this difference is that the reliability of the rated English affective variables is considerably lower than that of their Dutch counterparts. We therefore adjusted the correlations obtained in Studies 1 and 2 with a correction for attenuation (Spearman, 1904). This was done by dividing the obtained correlations by the square root of the product of the reliability estimates of the human judgments and the reliability of the predicted ratings (the reliability of the predicted ratings was set at one). This resulted in correlations that were virtually identical: .92, .85, .86 for valence, arousal, and dominance for the Dutch association data in Study 1, and .91, .83, .85 for the English data in Study 2.

**Table 3:** Highest correlations (r), 95% confidence intervals (95% CI), sample size (N) for each variable using *k*-NN with their respective value of k (k). All cross-validation correlations use the leave-one-out principle.

	*N*	*r*	95% CI	*k*

Valence	8770	.86	(.86–.87)	24
Arousal	8770	.69	(.68–.70)	44
Dominance	8770	.75	(.74–.76)	25
AoA	10032	.59	(.58–.61)	26
Concreteness	10957	.87	(.86–.87)	8

The best predictions for concreteness were obtained with k = 8, which resulted in a correlation with the human concreteness ratings of .87 (see Table 3). This was the same as the .87 correlation using Dutch data in Study 1, even when taking reliability into account. The Spearman-Brown corrected split-half correlations for concreteness in Study 1 fell in between .91 and .93 (5 lists of ca. 6,000 words), the correlation between the ratings of the overlapping words in Brysbaert, Warriner, et al. (2014) and the MRC database. (Coltheart, 1981) was .92. The predicted ratings for AoA had the lowest correlation with human ratings amongst the variables tested: .59 (k = 26). This was considerably lower than the .71 from Study 1, even after correcting for attenuation (.62 vs. .72).

**Parts 2a and 2b.** In order to compare our association-based approach to the text-based approach in Bestgen and Vincze (2012) and Recchia and Louwerse (2015), two additional analyses were conducted. The first analysis, comparing the association-based results with the findings of Bestgen and Vincze, is shown in Table 4. The correlations obtained based on the association data were considerably higher than those reported by Bestgen and Vincze (.71, .56, and .60 for valence, arousal, and dominance). All differences were significant (*p* < .001) using the Fisher’s *z* test for significance (Fisher, 1925).

**Table 4:** Highest correlations (r), 95% confidence intervals (95% CI), sample size (N) for each variable using *k*-NN with their respective value of k (k), for the ANEW (Bradley & Lang, 1999) norms. All cross-validation correlations use the leave-one-out principle.

	*N*	*i*	95% CI	*k*

Valence	946	.92	(.91–.93)	11
Arousal	946	.74	(.71–.77)	10
Dominance	946	.83	(.81–.85)	10

The second analysis compared the association model with the text-based one of Recchia and Louwerse (2015). The obtained correlations with the human ratings are shown in the second column of Table 5. The analysis was very similar to the one from Recchia and Louwerse (2015), with our test set being slightly smaller and our training set being considerably smaller. Recchia and Louwerse report correlations with human ratings of about .74, .57, and .62 for valence, arousal, and dominance. In this second analysis as well, we find that the values obtained with the association data are significantly (*p* < .001) higher.

**Table 5:** Highest correlations (r), 95% confidence intervals (95% CI), sample size (N) for each variable using *k*-NN with their respective value of k (k). Data is trained on the Warriner et al. (2013) norms, and tested with the ANEW (Bradley & Lang, 2017) norms.

	*N*	*r*	95% CI	*k*

Valence	2156	.89	(.88–.89)	13
Arousal	2156	.71	(.68–.73)	24
Dominance	2156	.76	(.74–.77)	23

To summarize, Study 2 replicates the findings of Study 1 using English instead of Dutch data. Valence, arousal, dominance, and concreteness, and to a lesser extent AoA, were predicted accurately, allowing us to again conclude that these variables are well embedded in a semantic model based on word associations. The correlations we obtained for the affective variables, making use of solely a pairwise similarity measure and a simple *k-*NN approach, are the highest reported in the literature to our knowledge.

## General Discussion

Mental representations derived from word associations straightforwardly account for valence, dominance, and arousal. Using the average valence, dominance, and arousal value of the *k*-nearest neighbors of a word in an association corpus, its own valence, dominance, and arousal can be reliably approximated, resulting in correlations with human ratings above .90 (for valence) and around .85 (for dominance and arousal), after correction for attenuation, in both Dutch and English. Word associations also predict the concreteness of words, another semantic variable on which the words vary widely. Predictions derived from this model accounted for direct participant ratings for these four variables well or very well.

Several studies conducted over the past few years showed that word associations are able to predict pairwise semantic relatedness and similarity judgments rather accurately (De Deyne et al. 2013) and that they also predict the results of a triadic comparison task (De Deyne, Navarro, Perfors, & Storms, 2016). In this study, we extended these findings by showing that they can also clearly account for general affective and lexico-semantic characteristics. Moreover, by demonstrating the clear presence of the affective dimensions and the concreteness distinction in the association-based representation, our findings argue against ignoring these dimensions when studying word meaning, as is often done in the literature on semantic concepts (Murphy, 2002).

The fifth variable studied in this paper, the (estimated) age at which words are learned, could be predicted only modestly, with predictive correlations around .60 for English and .70 for Dutch. One may wonder why AoA lags behind the affective dimensions and concreteness. While previous work has established independent effects for AoA even when concreteness and word frequency are considered, it is quite possible that apart from a semantic locus (see Brysbaert & Ghyselinck, 2006) AoA is also determined by non-semantic aspects of language. The deviating nature of AoA as compared to the affective variables and concreteness also shows in the finding that word co-occurrence models yield worse predictions for AoA than for the other variables of interest. We do want to stress, though, that the modest association-based prediction of AoA was not worse than the best prediction of that variable based on word co-occurrence models.

Norms often contain additional information about variance across raters. For example, the gender, age, and education level of raters can all have an effect, resulting in norms that differ significantly between groups that vary on these characteristics (see Warriner et al. 2013). In principle, word associations could account for these differences when information about the participants that generated the associations is available. In other words, word associations might be collected from specific groups of people (democrats, republicans, men, women; see Szalay & Deese, 1978) and used to obtain group-specific predictions of lexical norms. Currently, we are gathering word association data in clinical groups (such as depressed and schizotypy patients) to see if such syndrome-specific data can capture the language-specific behavior of these patient groups.

### Are word associations an alternative to word co-occurrences?

It is fair to say that natural language models derived from word co-occurrences currently constitute the dominant approach to study semantic systems that cover broad semantic areas (Bullinaria & Levy, 2007, 2012; Fisher, 2010; Hollis & Westbury, 2016). The wide accessibility of the internet provided opportunities to develop an alternative to these models by using crowd sourcing to gather vast numbers of word associations. In the two studies described in this paper, the association-based model was not only shown to account well for the affective and lexico-semantic variables that we studied, its predictions clearly outperformed those of the co-occurrence models. This was shown in Study 2 through indirect comparisons with results from recent studies in English where valence, arousal, and dominance were predicted from state-of the-art text-based word co-occurrence models (Bestgen & Vincze, 2012; Mandera et al. 2015; Recchia & Louwerse, 2015), but also in a direct comparison in Study 1 with Dutch material, where predictions based on associations and on word co-occurrences were compared in the fairest possible way, using the same criterion variables and the same statistical prediction methods.

We do acknowledge the fact that the association-based model we use is based on human judgments, just like the lexico-semantic norms we predict. Some shared processes (e.g., memory retrieval) might be encoded (partly) in the association-based model, and this might be an advantage word associations have over co-occurrence models that derive semantic structure from naturally occurring language (Jones et al. 2015). We do think, however, that this is not the only reason why word associations do better because, for instance, one would expect the same advantage for predicting concreteness ratings, while these ratings were predicted equally well using the word co-occurrence and the association model (Study 1). We propose to take a dialectic approach, where the processes involved in word associations need to be explained, but also where word association data provide us with important information about mental representations. For instance, in a recent study, De Deyne, Navarro, Collell, and Perfors (2018) found that the addition of visual and affective features improved the relatedness predictions, for both concrete concepts compared at the basic level (e.g. apples – pears) and abstract concepts (frustration – envy), of text-based models but not association-based models, suggesting that word associations capture other types of properties (grounded in affect and imagery) than text.

Although the model used in Study 1 already moved beyond mere co-occurrences and used syntactic word dependencies to derive similarities, it is possible that the model can still be improved. Yet, the specific variant of the text-based co-occurrence model might not be as important as often assumed. This follows from the finding that similarity predictions from more recent lexico-semantic models based on neural networks, like word2vec (Mikolov et al. 2013), do not differ strongly from PPMI models like those described in Recchia and Louwerse (De Deyne, Perfors, & Navarro, 2017; Levy & Goldberg, 2014).

Because word associations consistently outperform language-based models on these tasks as well (De Deyne et al. 2013, 2016), it is quite likely that the ability of word associations to better capture relatedness than language-based models do explains its advantage over text-based approaches in predicting lexico-semantic variables such as valence and age of acquisition. Still, Study 1 showed that a small but significant part of the variance in the affective ratings and a more substantial part of the variance in the concreteness and the AoA ratings that could not be explained by the word association data *can* be accounted for by the language-based model. In other words, there is information in text corpora that is not captured in word associations, rending both approaches complimentary to some extent.

## Data Availability

No new data were collected during this research. The word association data can be requested at http://smallworldofwords.org/project/research. The ratings used in these studies are available as separate supplementary files to the article by Moors et al. (2013; https://doi.org/10.3758/s13428-012-0243-8), Brysbaert, Stevens, De Deyne, Voorspoels, and Storms (2014; https://doi.org/10.1016/j.actpsy.2014.04.010), Brysbaert, Warriner, and Kuperman (2014; https://doi.org/10.3758/s13428-013-0403-5), Kuperman, Stadthagen-Gonzalez, and Brysbaert (2012; https://doi.org/10.3758/s13428-012-0210-4), Warriner, Kuperman, and Brysbaert (2013; https://doi.org/10.3758/s13428-012-0314-x). The ANEW (Affective Norms for English Words; Bradley & Lang, 1999) can be requested at http://csea.phhp.ufl.edu/media/anewmessage.html.

## Footnotes

In a different set of analyses, not reported in this manuscript, using MDS and property fitting (i.e., the same method as in Vankrunkelsven et al. 2015) the co-occurrence-based correlation with AoA human ratings was higher, .73, than the one reported here. The difference with the equivalent association-based correlation, .72 was non-significant, *p* = .55.

We used 1,029 words instead of the 1,034 words used by Bestgen and Vincze (2012) because five words have newer ratings after the first update of the ANEW and we do not have access to the original dataset (1999).
